# Two Novel Multi-Functional Peptides from Meat and Visceral Mass of Marine Snail *Neptunea arthritica cumingii* and Their Activities In Vitro and In Vivo

**DOI:** 10.3390/md16120473

**Published:** 2018-11-27

**Authors:** Shan-Shan Zhang, Li-Wen Han, Yong-Ping Shi, Xiao-Bin Li, Xuan-Ming Zhang, Hai-Rong Hou, Hou-Wen Lin, Ke-Chun Liu

**Affiliations:** 1 Biology Institute, Qilu University of Technology (Shandong Academy of Sciences), Jinan 250103, China; qingshuibaikai@126.com (S.-S.Z.); hanlw@sdas.org (L.-W.H.); syp3317@163.com (Y.-P.S.); bin85666666@163.com (X.-B.L.); lenghanxing_0@163.com (X.-M.Z.); caomu_1314@163.com (H.-R.H.); 2 Shandong Provncial Engineering Laboratory for Biological Testing Technology, Key Laboratory for Drug Screening Technology of Shandong Academy of Sciences, Jinan 250103, China; 3 Research Center for Marine Drugs, State Key Laboratory of Oncogenes and Related Genes, Department of Pharmacy, School of Medicine, Shanghai Jiao Tong University, Shanghai 200127, China; Franklin67@126.com

**Keywords:** *Neptunea arthritica cumingii*, multi-functional peptides, antioxidant activity, ACE-inhibitory activity, anti-diabetic activity

## Abstract

*Neptunea arthritica cumingii* (*Nac*) is a marine snail with high nutritional and commercial value; however, little is known about its active peptides. In this study, two multi-functional peptides, YSQLENEFDR (Tyr-Ser-Gln-Leu-Glu-Asn-Glu-Phe-Asp-Arg) and YIAEDAER (Tyr-Ile-Ala-Glu-Asp-Ala-Glu-Arg), were isolated and purified from meat and visceral mass extracts of *Nac* using a multi-bioassay-guided method and were characterized by using liquid chromatography-tandem mass spectrometry. Both peptides showed high antioxidant, angiotensin-converting enzyme (ACE)-inhibitory, and anti-diabetic activities, with half-maximal effective concentrations values less than 1 mM. Antioxidant and ACE-inhibitory activities were significantly higher for YSQLENEFDR than for YIAEDAER. In a zebrafish model, the two peptides exhibited strong scavenging ability for reactive oxygen species and effectively protected skin cells against oxidative damage without toxicity. Molecular docking simulation further predicted the interactions of the two peptides and ACE. Stability analysis study indicated that the two synthetic peptides maintained their activities under thermal stress and simulated gastrointestinal digestion conditions. The low molecular weight, high proportion of hydrophobic and negatively-charged amino acids, and specific C-terminal and N-terminal amino acids may contribute to the observed bio-activities of these two peptides with potential application for the prevention of chronic noncommunicable diseases.

## 1. Introduction

Marine taxa are rich in bioactive compounds [1] that show antioxidative, antihypertensive, anti-diabetic, antimicrobial, and antitumor bioactivities [2], and are thus potentially valuable for the prevention and treatment of chronic noncommunicable diseases (NCDs) [3,4]. Accordingly, recent research has focused on bioactive peptides isolated from marine organisms [5].

Mollusks are the second largest phylum of the animal kingdom. In addition to their ecological roles, they have great commercial value as food [6]. Mollusks also contain many potential active compounds for the development of dietary supplements, functional foods, nutraceuticals, and medicine [4,7]. However, little is known about these active compounds, and mollusks are a relatively undeveloped resource for high-value products.

*Neptunea arthritica cumingii* (*Nac*) is a large-sized predatory gastropod belonging to the family Buccinidae, which includes well-known scavengers [8]. It mainly lives in the sea at depths from 10 to 78 m in China, Japan, North Korea, and South Korea. *Nac* has high commercial value owing to its hypertrophic and tendermeat, delicious taste, and high nutritional value, but its low hatching rate limits productivity. Despite studies of its genome, auxanology, reproductive features [9,10,11,12], and nutritive composition [13,14], little is known about the active chemical composition of *Nac*. To date, only tetramine, histamine, and choline derivatives with neural activity have been isolated from *Nac* [15,16]. We previously reported peptide extracts from whelks that show ACE-inhibitory activity [17]. Hence, we hypothesized that *Nac* might also contain peptides with activities that have potential for the prevention of NCDs.

To explore this possibility, natural multi-functional peptides were isolated and purified from the meat and visceral mass extracts of *Nac* using a multi-bioassay-guided method. The amino acid sequences of two of the isolated peptides were identified with liquid chromatography–tandem mass spectrometry (LC-MS/MS). The antioxidant, ACE-inhibitory, and anti-diabetic activities were evaluated in vitro, and the bioactivity and toxicity were evaluated in vivo in a zebrafish model (*Danio rerio*). Owing to their rapid organogenesis, transparent embryos, and high genetic similarity with humans [18], zebrafish are used extensively for studies of human diseases and activity screening [19]. Special cells of nitroreductase (NTR)-expressing transgenic zebrafish are efficiently ablated after treatment with metronidazole (MTZ) [20], and reactive oxygen species (ROS ) are rapidly generated in specific tissues or cells [21]. Because ROS overproduction can induce apoptosis [22], antioxidants can promote the regeneration of ablated cells by effectively mitigating ROS generation [20,21]. Therefore, an MTZ-treated Tg (*krt4:NTR-hKikGR*)^cy17^ zebrafish in which the NTR-hKikGR fusion protein is overexpressed under control of the skin-specific *krt4* promoter [23] was used as an ideal model for studying ROS-related pharmaceutical interventions in vivo. In addition, the molecular mechanisms of interactions between angiotensin-converting enzyme (ACE) and ACE-inhibitory peptides were preliminarily explored using molecular docking. Finally, to examine the therapeutic feasibility, the stabilities of the synthetic peptides for maintaining 2,2-diphenyl-1-(2,4,6-trinitrophenyl) hydrazyl (DPPH) radical scavenging activity and ACE-inhibitory activity under thermal stress and exposure to gastrointestinal digestion were investigated.

## 2. Results

### 2.1. Proximate Analysis

The mean ± standard deviation length and width of the shell of *Nac* were 105.33 ± 2.39 mm and 63.17 ± 3.20 mm, respectively. The fresh weight was 103.23 ± 2.30 g, and the weights of the meat and visceral mass were 17.34 ± 3.90 g and 24.41 ± 2.86 g, respectively. The proximate compositions of the meat and visceral mass are summarized in Figure S1 of the Supplementary Materials. The meat contained higher ash and lower fat contents than those of the visceral mass, but there was no significant difference (*p* > 0.05) in protein content between the two samples, suggesting that the meat has better nutritional quality than the visceral mass.

### 2.2. Bioassay-Guided Isolation of the Active Fraction

**Figure 1:**
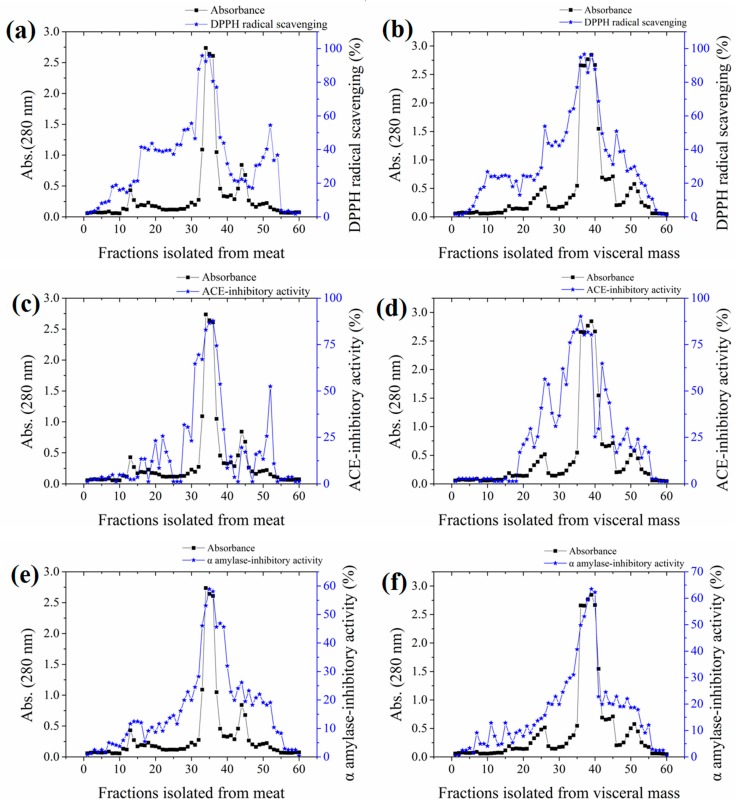
Absorbances and activities of fractions isolated from the meat and visceral mass of *Neptunea arthritica cumingii* (*Nac*) by using gel filtration column packed with Sephadex G25 gel. (**a**) the 2,2-diphenyl-1-(2,4,6-trinitrophenyl) hydrazyl (DPPH) radical scavenging activity, (**c**) angiotensin-converting enzyme (ACE)-inhibitory activity, and (**e**) α-amylase inhibitory activity of meat fractions; and (**b**) the DPPH radical scavenging activity, (**d**) ACE-inhibitory activity, and (**f**) α-amylase inhibitory activity of visceral mass fractions.

The yields of meat and visceral mass extracts were 5.02% and 5.15%, respectively. Fractions with stronger absorbance at 280 nm showed higher biological activities (Figure 1). Significant correlations (Pearson correlation coefficients ranging from 0.766 to 0.942) were observed in the antioxidant, ACE-inhibitory, and α-amylase inhibitory activities among fractions (Table S1 in the Supplementary Materials). The most active fractions 34–36 (M-F) for meat and 37–39 (VM-F) for visceral mass had 2,2-diphenyl-1-(2,4,6-trinitrophenyl) hydrazyl (DPPH) radical scavenging and ACE-inhibitory activities exceeding 80% and α-amylase inhibitory activity exceeding 50%, and they were thus collected for further investigation.

### 2.3. Molecular Weight Distribution

The molecular weight (MW) profiles of the M-F and VM-F each showed a major peak (more than 90%) in the low-molecular-mass region (Figure S2 in the Supplementary Materials). The average MW of the two major peptides was below 2000 Da (calibration curve molecular weight = 17.50–0.089T, R^2^ = 0.9785, where T is the retention time).

### 2.4. Amino Acid Profile of Active Fractions

Both M-F and VM-F had high contents of hydrophobic and positively/negatively charged amino acids (Table 1). In addition, significant differences between M-F and VM-F were observed with respect to the hydrophobic and aromatic amino acid contents (*p* < 0.01). M-F had higher contents of these amino acids.

### 2.5. Bioassay-Guided Purification of Active Peptides

**Figure 2:**
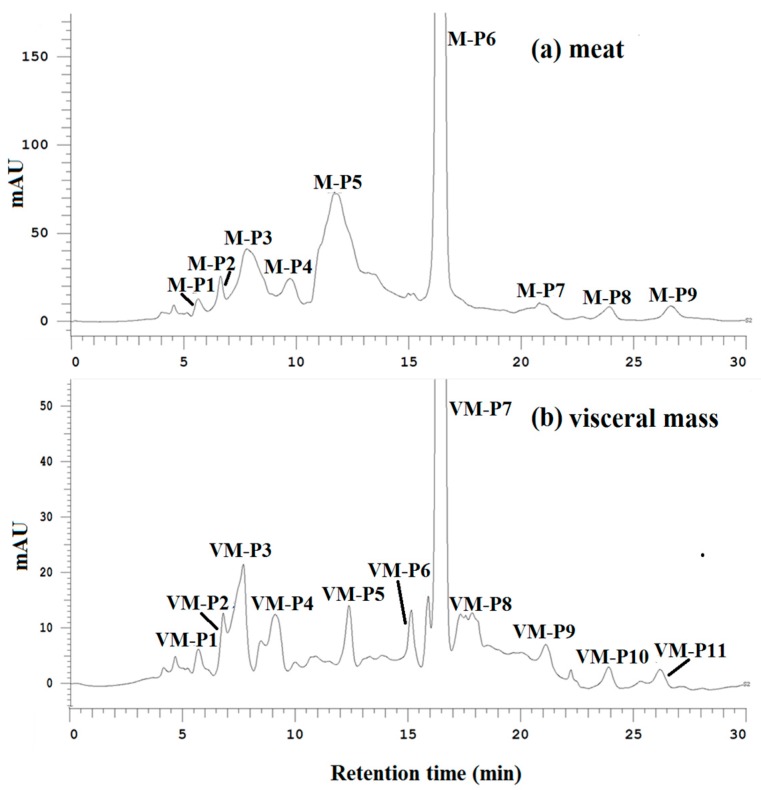
(**a**) Elution profiles of active fractions from the meat of *Nac* by hydrophilic interaction chromatography (HILIC) at 214 nm; (**b**) elution profiles of active fractions from the visceral mass of *Nac* by HILIC at 214 nm.

The DPPH radical scavenging, ACE-inhibitory and α-amylase inhibitory activities of all purified fractions were determined at the same concentration. As shown in Figure 2a,b, peptides M-P6 and VM-P7 were the main components of the meat and visceral mass. M-P6 and VM-P7 had the strongest activities overall (Table 2), and were thus considered the main active peptides of the meat and visceral mass, respectively, which were subject to further structure characterization and activity determinations.

### 2.6. Amino Acid Sequence of Active Peptides

A 10-residue peptide, YSQLENEFDR (Tyr-Ser-Gln-Leu-Glu-Asn-Glu-Phe-Asp-Arg), with a molecular weight of 1299 Da was identified from M-P6 (see the MS/MS spectra in Figure S3A of the Supplementary Materials) according to MS^2^ spectra, and an eight-residue peptide, YIAEDAER (Tyr-Ile-Ala-Glu-Asp-Ala-Glu-Arg), with a molecular weight of 965 Da was identified from VM-P7 (see MS/MS spectra in Figure S3B of the Supplementary Materials). The sequences of the two peptides were searched against the BIOPEP database [24], and no reports of the bioactivity of these peptides were found. Hence, the bioactivities of these two active peptides obtained from *Nac* were further investigated through in vitro and in vivo experiments.

### 2.7. Analyses of Active Peptide Activity

#### 2.7.1. In Vitro Antioxidant Activity

**Figure 3:**
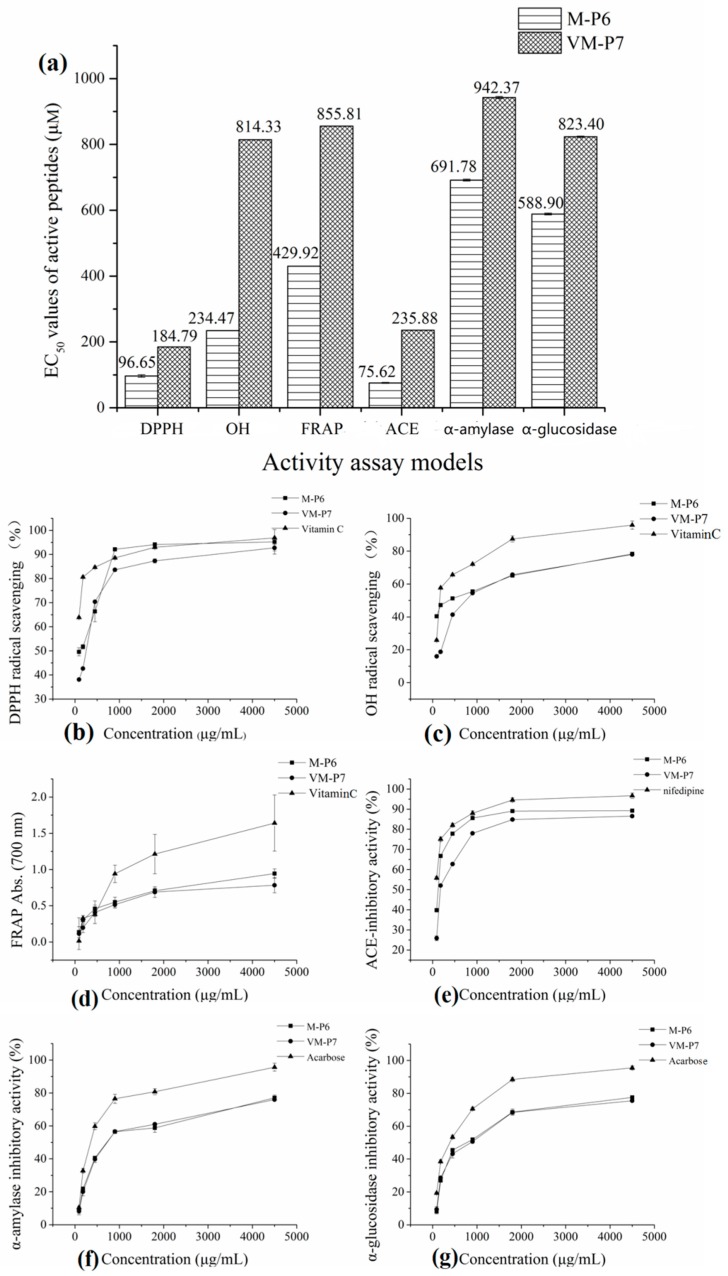
Bioactivities of two peptides. (**a**) half maximal inhibitory concentration (EC_50_) values in different models (** *p* < 0.01 compared with the VM-P7), (**b**) DPPH radical scavenging activity, (**c**) OH radical scavenging activity, (**d**) reducing power, (**e**) ACE-inhibitory activity, (**f**) α-amylase inhibitory activity, and (**g**) α-glucosidase inhibitory activity.

According to the half maximal inhibitory concentration (EC_50_) values of M-P6 and VM-P7 in three antioxidant assay models (Figure 3a), both two peptides showed high antioxidant activity. Furthermore, although that of M-P6 was significantly higher (*p* < 0.01). The antioxidant activities of M-P6 and VM-P7 showed a concentration-dependent increase from 90 to 4500 μg/mL (Figure 3b–d). At concentrations greater than 1 mg/mL (corresponding to 0.77 mM and 1.04 mM for M-P6 and VM-P7, respectively), the two peptides exhibited similar activity to that of vitamin C.

#### 2.7.2. ACE-Inhibitory Activity

Significantly greater ACE-inhibitory activity was also observed for M-P6 than for VM-P7 (*p* < 0.01, Figure 3a). Furthermore, the ACE-inhibitory activities of the two peptides increased within increasing concentrations but always less than nifepine (Figure 3e).

#### 2.7.3. Anti-Diabetic Activity

The two peptides showed similar anti-diabetic activity (Figure 3a, *p* > 0.05). Compared to the control (acarbose), the α-amylase and α-glucosidase inhibitory activities of the two peptides were relatively poor; however, activities of the two peptides increased as the concentration increased (Figure 3f,g).

#### 2.7.4. In Vivo Antioxidant Activity in Zebrafish Embryos

We next explored the effects of the two active peptides obtained from *Nac* on the mortality and morphology of zebrafish larvae. No death or malformation was observed after exposure to high, medium, and low concentrations of active peptide for 24 h.

For Tg (*krt4:NTR-hKikGR*)^cy17^ zebrafish, MTZ treatment can lead to ROS overproduction, apoptosis of skin cells, and reduction of fluorescence spots on skin; thus, this model is used to assess ROS scavenging capacity of sample by measuring the growth rate of fluorescent spots (FS) on the skin of Tg (*krt4:NTR-hKikGR*)^cy17^ transgenic zebrafish. Sample with antioxidant activity can remove ROS from transgenic zebrafish and prevent the skin cell apoptosis. Hence, an increased number of FS are observed.

**Figure 4:**
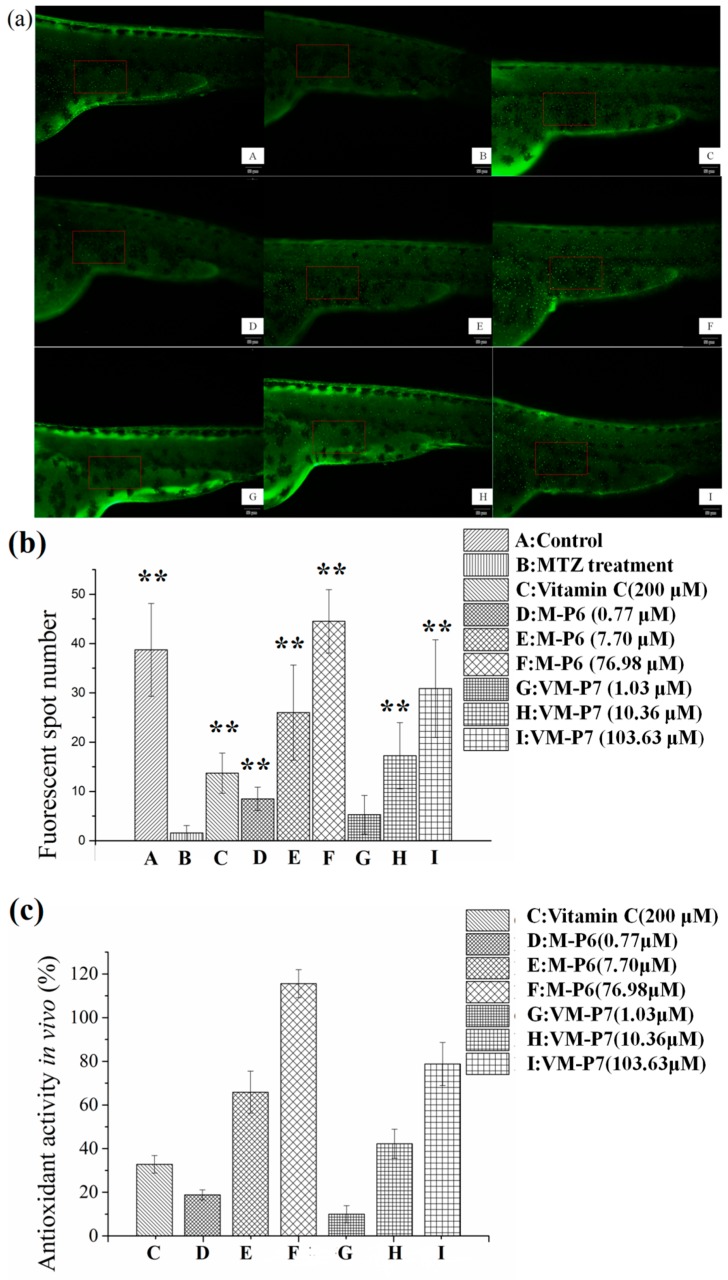
In vivo antioxidant activity of active peptides in zebrafish embryos (*n* = 10, mean ± standard deviation). (**a**) In vivo visualization of zebrafish skin fluorescence treatment with vehicle (A), metronidazole (MTZ; (B)), vitamin C (C), and low (D), medium (E), and high (F) concentrations of M-P6, as well as low (G), medium (H), and high (I) concentrations of VM-P7; (**b**) FS number statistic results of all groups; (**c**) In vivo antioxidant activity of all samples. ** indicates significant differences compared with the MTZ treatment group (*p* < 0.01).

According to visualization of zebrafish skin fluorescence results in Figure 4a, more FS were observed for the sample groups (Figure 4a(D–I)) than the negative control group incubated with MTZ but without peptides (Figure 4a(B)), even at a low concentration. Moreover, the FS number of all samples except VM-P7 at a low concentration showed significant difference (*p* < 0.01) compared with negative control (Figure 4b). There was no significant difference in the number of FS between the VM-P7 at a low concentration and negative control (Figure 4b(G,B)). Furthermore, more FS were detected for M-P6 than VM-P7 at medium and high concentrations (*p* < 0.01).

As shown in Figure 4c, the in vivo antioxidant activities of two peptides gradually increased with the increase of concentration. M-P6 showed better antioxidant activity compared with VM-P7. These results were consistent with the findings of in vitro antioxidant activity. In addition, the antioxidant activities of the two peptides at medium and high concentrations were significantly greater than that of the positive control vitamin C at 200 μM (*p* < 0.01). In addition, compared to the control group (no MTZ or peptides), more FS were detected for M-P6 at high concentrations (Figure 4b(F)). The reason for this result may be superior ROS scavenging ability of M-P6 promoting more skin cells regeneration. These results indicated that the two peptides could protect the skin cells of transgenic zebrafish against oxidative damage caused by MTZ.

### 2.8. Molecular Docking Simulation

**Figure 5:**
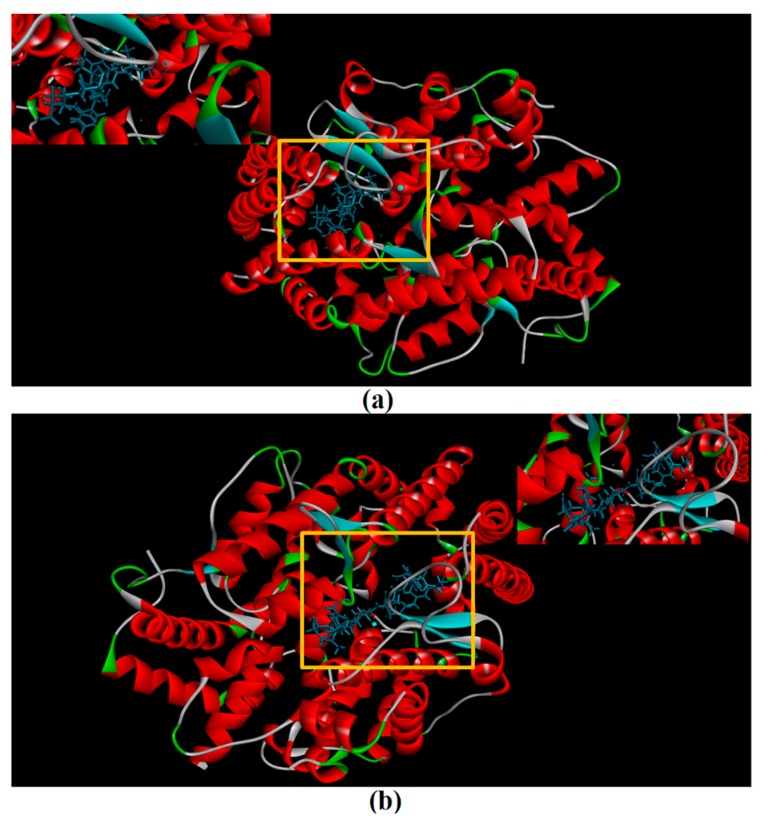
The molecular docking of YIAEDAER (**a**) from visceral mass and YSQLENEFDR (**b**) from meat to angiotensin-converting enzyme.

**Figure 6:**
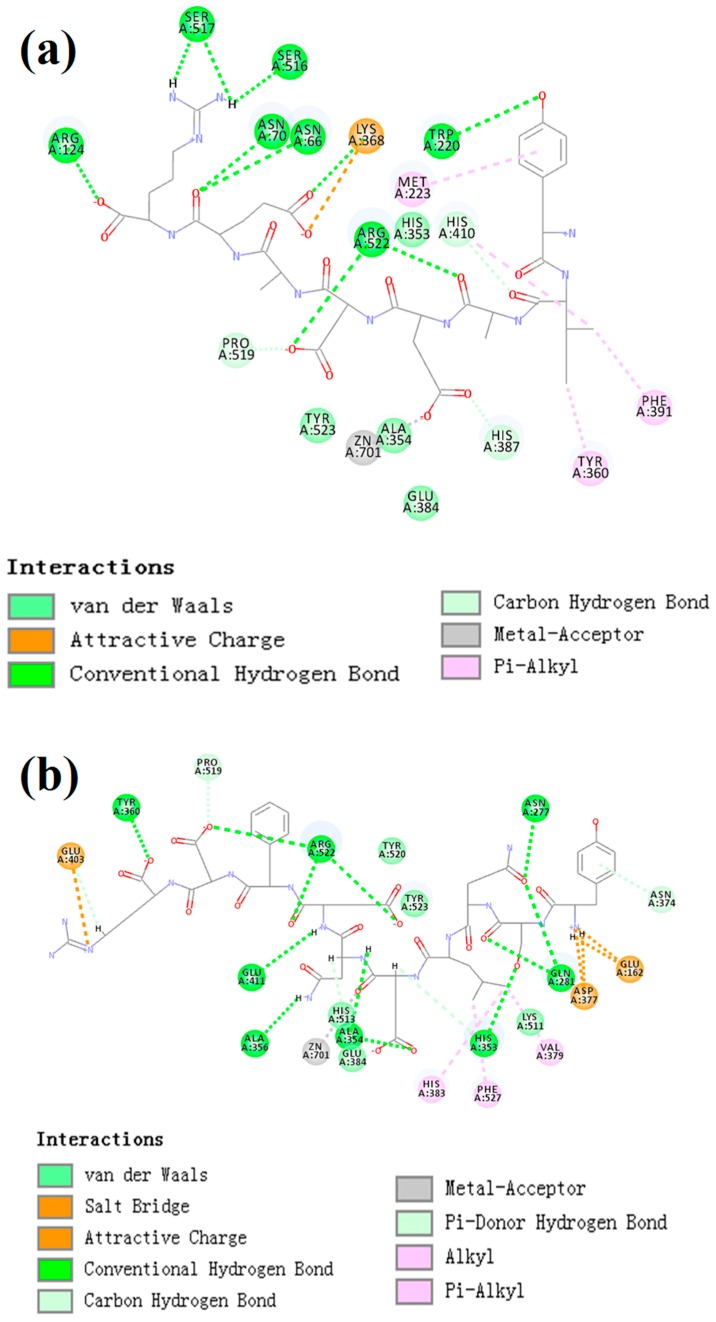
The two-dimensional diagram of molecular docking simulation of YIAEDAER (**a**) from visceral mass and YSQLENEFDR (**b**) from meat to angiotensin-converting enzyme.

CDocker was used to further investigate the intermolecular interactions between the active peptides and ACE. Both peptides showed a stable docking structure with ACE according to the—CDocker energy and—CDocker interaction energy values (Table 3). However, YSQLENEFDR displayed better binding affinity with ACE than YIAEDAER. The docking simulation results of two peptides are shown in Figure 5 and Figure 6. Both peptides successfully entered the channel of ACE after docking (Figure 5a,b). As shown in Figure 6a,b, both active peptides combined with ACE residues through van der Waals interactions, hydrogen bonds, and electrostatic, hydrophobic, and miscellaneous interaction forces. YIAEDAER made contract with the ACE residues His 353, Ala 354, Glu384, and Tyr523 via van der Waals interactions and formed 11 hydrogen bonds with ACE residues Asn66, Asn70, Arg124, Trp220, Lys368, His387, His410, Ser516, Ser517, Pro519, and Arg522, respectively. Electrostatic force were observed between YIAEDAER and the ACE residue Lys368 interacted through an electrostatic force, whereas interactions with Met223, Tyr360, Phe391, and His410 were through the hydrophobic force. YSQLENEFDR made contact with ACE residues Glu384, Lys511, His513, Tyr520, and Tyr523 via the van der Waals force, and formed 13 hydrogen bonds with ACE residues Glu162, Asn277, Gln281, His 353, Ala354, Ala356, Tyr360, San374, Asp377, Glu403, Glu411, Pro519, and Arg522, respectively. YSQLENEFDR and ACE residues Glu162, Asp377, and Glu403 interacted with electrostatic force, whereas a hydrophobic force formed between YSQLENEFDR and ACE residues Val379, His383, and Phe527. Both YIAEDAER and YSQLENEFDR interacted with Zn (II) via miscellaneous force.

### 2.9. Stablity of Synthetic Peptides against Thermal and Gastrointestinal Digestion Treatments

A series of samples with concentration range from 90 μg/mL (corresponding to 69.28 μM and 93.26 μM for YSQLENEFDR and YIAEDAER, respectively) to 1800μg/mL were used in stability analysis. EC_50_ values (expression in molar concentrations) of active peptide under different treatment conditions were used as evaluation standard.

**Figure 7:**
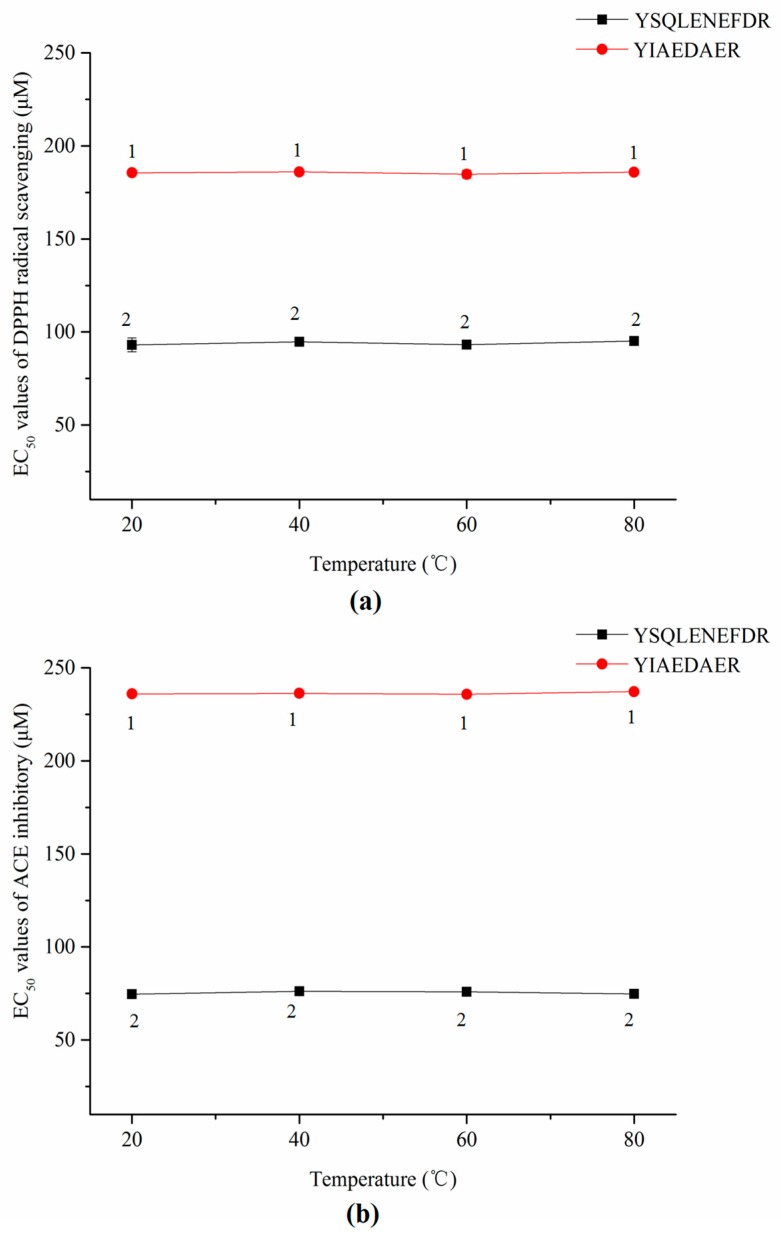
EC_50_ values of DPPH radical scavenging (**a**) and ACE inhibitors (**b**) of two synthetic peptides treated with thermal treatment. The same numbers means no significantly different in a group (*p* > 0.05).

As shown in Figure 7a,b, the thermal treatments did not significantly affect the DPPH radical scavenging activities or ACE inhibitory activities of both synthetic peptides according to a lack of significant change in EC_50_ values (*p* > 0.05).

**Figure 8:**
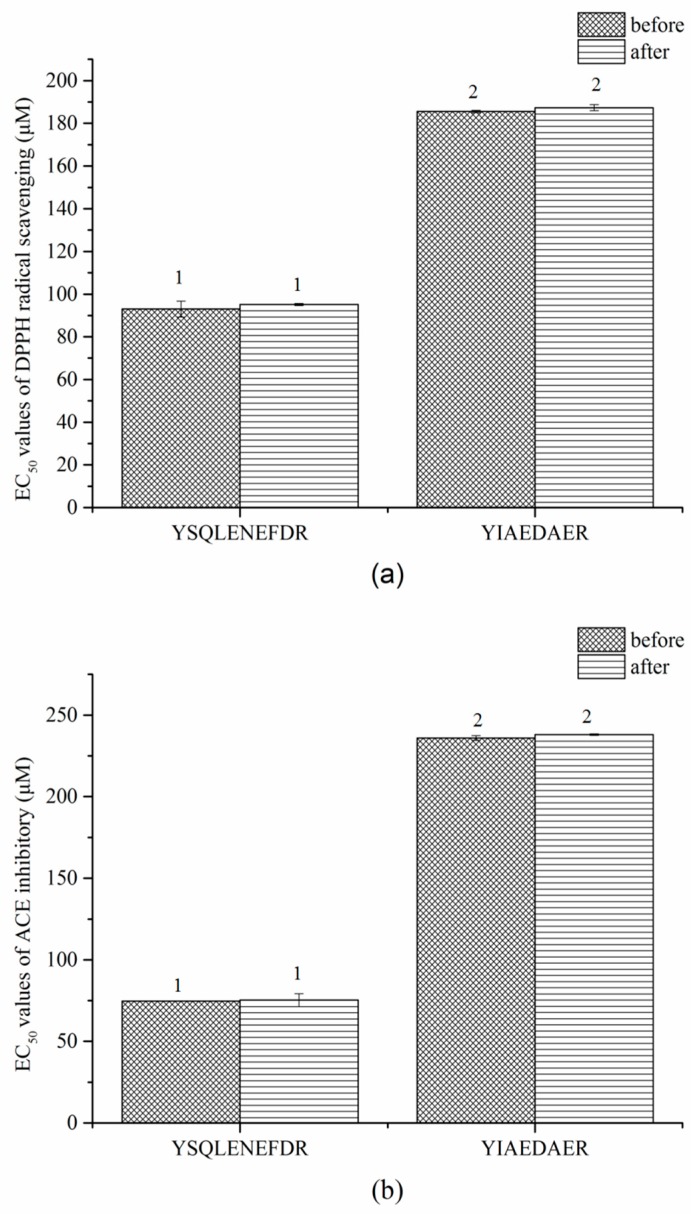
EC_50_ values of DPPH radical scavenging (**a**) and ACE inhibitors (**b**) of two synthetic peptides treated with gastrointestinal digestion treatment. The same numbers indicate no significant difference in a group (*p* > 0.05).

Similar results were observed in the gastrointestinal digestion treatment (Figure 8a,b). The EC_50_ values of DPPH radical scavenging and ACE inhibitory of both peptides slightly increased after digestion but the difference was not significant (*p* > 0.05). These results indicated that the two peptides, YSQLENEFDR and YIAEDAER, have thermal and gastrointestinal stability.

## 3. Discussion

Two novel multi-functional peptides, YSQLENEFDR and YIAEDAER, were isolated, purified and identified from the meat and visceral mass of *Nac* under multi-bioassay guidance using gel filtration chromatography, reversed phase-high-performance liquid chromatography (RP-HPLC), hydrophilic interaction chromatography (HILIC), and LC-MS/MS. The total proportions of hydrophobic and negatively charged amino acids were relative high in sequences of both peptides. Both peptides showed strong in vitro antioxidant, ACE-inhibitory, and anti-diabetic activities, along with potent scavenging ability for ROS and protected skin cells against oxidative damage in a zebrafish model.

These high antioxidant activities of the two peptides could be attributed to their low molecular weight, which facilitates access to oxidant-antioxidant systems [25]. However, the amino acid composition and peptide sequence are also important determinants of antioxidant activity. Peptides of freshwater mussels with a high molecular weight were reported to show relatively high antioxidant activity due to the abundant hydrophobic amino acids [26]. Indeed, hydrophobic and aromatic amino acids are known to play important roles in the antioxidant activity of peptides [27]. Negatively charged amino acids have strong antioxidant effects because the excess electrons are free to interact with free radicals [28]. The position of Tyr at the N terminus and the dipeptide Glu-Leu and Ala-Glu of the peptide sequence can also contribute to antioxidant activity [29]. Moreover, Glu in the peptide sequence increases antioxidant activity via promoting oxidized glutathione production [30]. Therefore, the higher antioxidant activity of YSQLENEFDR may be due to the presence of aliphatic and aromatic amino acids (Tyr, Ser, Leu and Phe), especially Tyr at the N terminus and Glu-Leu, as well as the acidic amino acid Glu. Similarly, the hydrophobic amino acids (Tyr, Ile, Ala) and the acidic amino acid Glu also contributed to the antioxidant activity of YIAEDAER.

ACE catalyzes the conversion of angiotensin I to angiotensin II, a potent vasoconstrictor, and promotes degradation of the vasodilator bradykinin [31]; thus, inhibition of ACE activity may help with reducing blood pressure. Because high-molecular-weight peptides cannot occupy the active site of ACE [32], ACE-inhibitory peptides are typically 2–30 amino acids in length [33]. Moreover, peptides with high aromatic acid contents have been shown to have higher ACE-inhibitory activity [34]. Peptides containing Arg at the C terminus exhibit inhibitory activity due to the positive charge of the guanidine group [35]. Tyr at the N terminus can enhance the activity. This is supported by data recorded in biopep database. Four peptides, YPR, YLYEIAR, YLYEIARR, and YIPIQYVLSR, which possess similar amino acid residues with active peptides from *Nac*, show EC_50_ values of 16.5, 16.00, 86.00 and 132.50 μM. Furthermore, Asn potentially contributes to ACE-inhibitory activity [36]. Hence, Arg in the C terminus, Tyr in the N terminus and the aromatic amino acid residues in YSQLENEFDR and YIAEDAER might explain their potent ACE-inhibitory activities. In addition, the higher inhibitory activity of YSQLENEFDR than YIAEDAER may be attributed to the presence of the Asn residue.

α-Amylase and α-glucosidase are key enzymes for starch and oligosaccharide digestion [37], and their inhibition is an effective method for controlling glucose homeostasis in diabetic patients [38]. Low-molecular-weight peptides (<3 kDa) have potent inhibitory activities of these digestive enzymes [39]. Aromatic and hydrophobic amino acid residues also play important roles in α-amylase and α-glucosidase inhibitory activities [40,41]. Accordingly, the inhibitory activities of YSQLENEFDR and YIAEDAER could be attributed to their low molecular weights along with the presence of Tyr, Glu, and Arg residues.

Moreover, both purified peptides showed good ROS-scavenging ability in zebrafish in vivo with no toxic effects, and could effectively prevent the skin cell damage caused by peroxidation.

There is no detailed information on the molecular mechanisms of interactions between ACE and ACE-inhibitory peptides from *Nac*. Therefore, to facilitate further research and development of active peptides, molecular docking simulation was conducted between ACE and active peptides from different parts of *Nac* as a preliminary analysis using CDOCKER.

ACE contains three main active site pockets: S1, S2, and S1′. The S1 pocket includes the residues Ala354, Glu384, and Tyr523; the S2 pocket contains Gln281, His353, Lys511, His513, and Tyr520; and S1′ contains Glu162 [42]. The molecular docking results clearly showed interactions of the active peptides with these active site residues of ACE, thus contributing to their inhibition activities. YIAEDAER establishes interactions with the S1 pocket (Ala354, Glu384, and Tyr523) and S2 pocket (His 353) of ACE via van der Waals forces, whereas YSQLENEFDR establishes interactions with all active site residues via van der Waals interactions, hydrogen bonds, and electrostatic forces. Moreover, both peptides established hydrogen bonds with the Arg522 of ACE, which has been reported as an imported residue for activity of the enzyme [43]. Addition, both peptides directly interacted with Zn^2+^ at the ACE active site via a miscellaneous force, which likely promoted the ACE-inhibitory activities of peptides since previous work has shown that interactions between ACE inhibitors and Zn^2+^ play an important role in deactivating ACE [44]. Furthermore, more hydrogen bonds were formed between YSQLENEFDR and ACE, and the number of hydrogen bonds plays a major role in determining interactions between inhibition peptides and ACE [45]. Overall, these results indicate that YSQLENEFDR exhibit better ACE inhibition activity, which is attributed to its more effective interaction with the active sites, supporting the results of the in vitro ACE inhibition assay of the active peptides.

Since functional food or drug processing technology may involve thermal sterilization and drying, it is essential to confirm the stability of active peptides against thermal and gastrointestinal digestion treatments for their applications as functional foods or drugs. Our results suggest that the thermal processing technology may not affect the antioxidant and ACE activities of YSQLENEFDR and YIAEDAER. In vitro simulated gastrointestinal digestion is an effective initial assessment method for the bioavailability of active peptides prior to in vivo applications [46]. Although the antioxidant and ACE activities of YSQLENEFDR and YIAEDAER were slightly reduced after gastrointestinal digestion, both synthetic peptides still showed good activity levels, and the activities of YSQLENEFDR were still better than those of YIAEDAER. Furthermore, the two synthetic peptides showed similar bioactivities with respect to the EC_50_ values of natural peptides in DPPH radical scavenging and ACE-inhibitory activities. It is indicated that modification is an effective method to improve stability, boost bioavailability or enhance activities of natural peptides [47]. Thus, these two natural peptides can be used as precursors of peptides therapeutics.

Overall, the study indicates that two novel natural multi-functional peptides isolated from *Nac* show good antioxidant, ACE-inhibitory, and anti-diabetic activities in vitro, as well as strong ROS scavenging ability in vivo without toxicity. The ACE inhibition of the two peptides may be mainly attributed to the interaction with the active site residues of ACE and Zn^2+^. Furthermore, the DPPH radical scavenging and ACE-inhibitory activities of the two synthetic peptides were stable after thermal treatment (20–80°C) and gastrointestinal digestion. Thus, these two active peptides, YSQLENEFDR and YIAEDAER, have potential for the treatment and prevention of NCDs. For further study, we will focus on the mechanism of action of these peptides.

## 4. Materials and Methods

### 4.1. Materials

Live *Nac* samples (see Figure S4A in the Supplementary Materials) of similar sizes were purchased from a market in Jinan, China. The samples were washed with water and the moisture on the shell was removed by drying at 25 °C. The shell and operculum were removed, and the soft body (Figure S4B in Supplementary Materials) was separated into the meat (Figure S4C in Supplementary Materials) and visceral mass (Figure S4D in Supplementary Materials). The samples were freeze-dried, ground into a power, and stored at −20 °C until analysis.

### 4.2. Reagents and Animals

DPPH, angiotensin-converting enzyme (0.25 U·mL^−1^, from the rabbit lung), N-hippuryl-His-Leu hydrate (HHL), hippuric acid, and amino acid standards were purchased from Sigma-Aldrich (Shanghai, China). α-Glucosidase protease (50 U/mg, from yeast), α-amylase protease (50 U/mg, from *Bacillus subtilis*), acarbose, 4-nitrophenyl-β-d-galactopyranoside, 6-aminoquinolyl-N-hydroxysuccinimidyl carbamate (AQC), ribonuclease (13,700 Da, from the bovine pancreas), aprotinin hydrochloride (6511 Da, from the bovine lung), angiotensin II (1046 Da), and l-serine (105 Da) were purchased from Yuanye Biological Technology Co. (Shanghai, China). Trypsin (250 U/mg), pepsin (250 U/mg), Sephadex G25 gel, nifedipine, and vitamin C were purchased from Solarbio (Beijing, China). Transgenic zebrafish Tg (*krt4:NTR-hKikGR*)^cy17^ are maintained in our lab. All chemicals and reagents used for HPLC were of chromatographic grade and other chemicals and reagents were of analytical grade.

### 4.3. Zebrafish Maintenance and Embryo Handling

Adult zebrafish were maintained at 28 °C under a 14/10 h light/dark cycle and supplied with freshwater, aeration, and food. Embryos were obtained from natural spawning; they were collected within 30 min and cultured in an aquarium. The embryos were used within 24 h. The experiments were performed in accordance with standard ethical guidelines. The procedures were approved by the Ethics Committee of the Biology Institute of Shandong Academy of Science.

### 4.4. Proximate Composition

The proximate compositions of the two parts of *Nac*, including moisture, ash, crude protein, and fat contents, were determined according to the methods of the Association of Official Analytical Chemists [48].

### 4.5. Peptide Extraction

The ground meat and visceral mass of *Nac* were suspended in 50% (*v*/*v*) ethanol in ultrapure water (sample: solution = 1:10, *w*/*v*), and the pH was adjusted to 5.0 using acetic acid. The mixture was stirred continuously using a magnetic stirrer at 30 °C for 6 h. The suspension was centrifuged at 6000× *g* for 20 min. The supernatant was concentrated by rotary evaporation to remove ethanol at 40 °C, and then, an equal volume of hexane was added in a separating funnel three times to minimize low polarity interference. The lower layer was freeze-dried and stored at −20 °C until further separation.

### 4.6. Bioassay-Guided Isolation of Active Fractions

Lyophilized samples were dissolved in 10 mL of 0.01 mol/L HCl at 50 mg/mL. After filtering through a 0.45-μm syringe filter, 5 mL of the sample was loaded onto a gel filtration column packed with Sephadex G25 gel (1.8 cm × 60 cm) pre-equilibrated with 0.01 mol/L HCl. The column was eluted with 0.01 mol/L HCl at a flow rate of 15 mL/h. Fractions were collected every 5 mL and absorbance was measured at 280 nm to measure the in vitro antioxidant, ACE-inhibitory, and α-amylase inhibitory activities of all fractions. Most of the active fractions separated on Sephadex G25 column were freeze-dried for further analysis and purification.

### 4.7. Molecular Weight Distribution of Active Fractions

The molecular weight distribution of the active fractions obtained from the meat and visceral mass were determined by gel permeation chromatography using a TSK-gel G2000 SWXL column (7.8 mm × 250 mm) (TOSOH, Yamaguchi, Japan) according to previously reported methods [36], except the flow rate was set to 0.2 mL·min^−1^. The column was calibrated with ribonuclease, aprotinin hydrochloride, angiotensin II, HHL, and l-serine.

### 4.8. Amino Acid Compositions of Active Fractions

Active fractions were hydrolyzed according to previously reported methods [49]. After acid hydrolysis, samples and amino acid standards were derivatized with AQC and determined by RP-HPLC [50]. The amino acid compositions of the two fractions were identified and quantified from standard curves of amino acid mixtures. All samples were determined in triplicate.

### 4.9. Bioassay-Guided Purification of Active Peptides

Freeze-dried active fractions were dissolved in 10 mM ammonium acetate buffer (pH 6.0) at 1 mg·mL^−1^. Samples were filtered through a 0.45-μm microporous membrane and further separated on a Welch HILIC amide column (4.6 mm × 250 mm, 5 μm). The binary mobile phase composed of acetonitrile and 10 mM ammonium acetate buffer (pH 6.0) (80:20 *v*/*v*) was pumped at a flow-rate of 1 mL/min. The injection volume was 20 μL. The absorption peak was monitored at 214 nm and all absorption peaks were collected. The activities of all fractions were then determined. The most active fractions from the two sources were freeze-dried and stored at −20 °C for further identification.

### 4.10. Identification of Active Peptide Sequences by Nano-LC-LTQ-Orbitrap-MS/MS

The amino acid sequences of active peptides were identified using an EASY-Nlc1000 chromatography system (Thermo Finnigan, Bremen, Germany) coupled to an LTQ Orbitrap Velos Pro mass spectrometer (Thermo Finnigan) according to previously published methods [51], with some modifications. The purified peptides were resolved in ultrapure water with 0.1% trifluoroacetic acid at a concentration of 0.1 mg/mL. Then, 2 μL of the sample was injected into the trap column (100 μm × 20 mm, RP-C_18_; Thermo Inc.) for preconcentration. The preconcentrated sample was automatically entered into an analysis column (75 μm × 150 mm, RP-C_18_; Thermo Inc.). The sample was eluted with 0.1% *v*/*v* formic acid in ultrapure water for 60 min with a flow rate of 300 nL/min at 30 °C. Mascot 2.3 (Matrix Science, Boston, MA, USA) was used for data analysis. The NCBInr database was used for peptide identification with an expected value threshold of less than 0.05.

### 4.11. Determination of Activities

#### 4.11.1. DPPH Radical Scavenging Activity

The DPPH radical scavenging activity of the two peptide fractions was determined according to the methods described by Lee et al. [52]. Vitamin C was used as a positive control. The EC_50_ values for DPPH radical scavenging was determined.

#### 4.11.2. Ferric Reducing Capacity

The reducing power of the peptide fractions was assayed as described by Moayedi et al. [53]. Vitamin C was used as a positive control. The reducing power of active peptides was assayed by determining EC_50_ values.

#### 4.11.3. Hydroxyl Radical Scavenging Activity

The hydroxyl radical scavenging activity of peptide fractions was determined according to the methods described by Dong et al. [26]. Vitamin C was used as a positive control. The hydroxyl radical activity of active peptides was evaluated by EC_50_.

#### 4.11.4. Determination of Antioxidative Activity in Zebrafish Embryos

The fluorescence spots on the Tg (*krt4:NTR-hKikGR*)^cy17^ zebrafish skin are significantly reduced after MTZ-treatment due to excessive ROS production. The number of fluorescence spots on the skin will increase after incubation with antioxidant. Thus, we can evaluate the ROS scavenging ability of samples on the basis of changes in the number of fluorescence spots compared with the MTZ-treatment. The in vivo antioxidant activity of the active peptides was evaluated according to previously described methods [54] with some modification using the transgenic zebrafish line Tg (krt4:NTR-hKikGR)^cy17^. Twenty-four-hour-old transgenic zebrafish embryos were added to 24-well cell culture plates (10 embryos/well) and incubated with 2 mL of 10 mM MTZ (dissolved in fish water) and active peptides for 24 h at 28 °C. Zebrafish treated with fish water without MTZ and peptides were used as controls. Zebrafish treated with MTZ without peptides were used as negative controls. Vitamin C instead of peptides was used as a positive control. After incubation, zebrafish embryos were anesthetized with tricaine (0.16%, *w*/*v*), and fluorescence was observed using an FSX100 Bio Imaging Navigator instrument. FS were counted using ImagePro-Plus, and the in vivo antioxidant activity (%) of active peptides was determined by Equation 1.

Antioxidant activity (%) = {(FSs − FSnc)/(FSvc − FSnc)} × 100(1)

where FSs indicates the number of FS in the samples, FSnc indicates the number of FSin the negative control, and FSvc indicates the number of FS in the vehicle control.

#### 4.11.5. Determination of ACE-Inhibitory Activity

ACE-inhibitory activity was measured according to the methods of Chen et al. [55]. Nifedipine was used as a positive control. Results were reported as EC_50_ values.

#### 4.11.6. Determination of Anti-Diabetic Activity

*α*-Amylase and *α*-glucosidase inhibitory activities were assayed as described by Uraipong et al. [37]. Acarbose was used as a positive control. The inhibitory activity of active peptides was expressed as the EC_50_ value.

### 4.12. Peptide Synthesis

After identification by nano-LC-ESI-MS/MS, two active peptides (Tyr-Ser-Gln-Leu-Glu-Asn-Glu-Phe-Asp-Arg and Tyr-Ile-Ala-Glu-Asp-Ala-Glu-Arg) were synthesized by Cellmano Biotech Co. Ltd. (Hefei, China) with a purity of 99.02% and 96.43%, respectively.

### 4.13. Molecular Docking

In the docking study, human ACE was used as receptor. The crystal structure of ACE (1O8A.pdb) was obtained from the Protein Data Bank (https://www.rcsb.org/structure/1O8A). The 3D structure of active peptide was constructed and energy minimized using MM2 molecular mechanics method with Chem3D Pro 14.0 (CambridgeSoft Co., MA, USA). Before docking, the structure of ACE was removed water molecules and inhibitors, retained the cofactors zinc and chloride atoms and cleaned protein. Then, the ACE and two active peptides were energetically minimized by the CHARMm force field, respectively. The automated molecular docking studies of the active peptides at the ACE binding sites were performed using the CDOCKER module according to the method described by Deng et al. [45]. The binding site sphere was set as x:40.302, y:37.243 and z:48.948 with radius of 20 Å. Evaluation of the molecular docking was performed according to values of -CDocker energy and -CDocker interaction energy. The best conformation of peptide and ACE showed the highest values of -CDocker energy and -CDocker interaction energy.

### 4.14. Stability against Thermal and Gastrointestinal Digestion Treatments

Thermal stability and gastrointestinal digestion stability of two synthetic peptides were determined according to Chen et al. [56]. The incubated temperature for peptide solutions was set as 20, 40, 60, and 80 °C, respectively. DPPH radical scavenging activities and ACE inhibitory activities of the peptide solutions were measured as the above description.

### 4.15. Statistical Analysis

All tests were repeated three times and results are presented as means ± standard deviation. SPSS 16.0 (SPSS Inc., Chicago, IL, USA) was used for statistical analyses. All figures were generated using Origin 9.0 (Origin Lab, Northampton, MA, USA). One-way analysis of variance was used to analyze differences among groups; *p* < 0.05 was considered statistically significant. Pearson correlation coefficients were used to evaluate correlations among contents and activities. The molecular docking was evaluated and analyzed by Discovery Studio 2.5 (DS2.5, Accelrys, San Diego, CA, USA) and Discovery Studio 4.5 Visualizer (DS4.5, Accelrys, San Diego, CA, USA).

## 5. Patents

(1) Zhang, S.S.; Liu, K.C.; Han, L.W.; Zhang, X.M.; Li, X.B.; Zhang, Y. Preparation and Application of Peptides with the Function of Repairing Oxidative Damage. Patent No. 201810915407.5.

(2) Han, L.W.; Zhang, S.S.; Liu, K.C.; Li, X.B.; Zhang, X.M.; Hou, H.R; Sun, C. Preparation and Application of Peptides with the Function of Preventing Cardio-Cerebrovascular Disease. Patent No. 201810916171.7.

## Figures and Tables

**Table 1:** Amino acid compositions of active fractions (*n* = 3, mean ± standard deviation (SD)).

Amino Acid	Meat (g/kg)	Visceral Mass (g/kg)
asp	42.24 ± 7.69	73.39 ± 9.67 **
glu	99.87 ± 5.60	65.21 ± 6.73 **
ser	21.82 ± 1.09	24.55 ± 5.99 *
gly	30.65 ± 3.15	36.29 ± 7.46 *
his	31.12 ± 1.03	10.90 ± 3.84 **
arg	388.77 ± 5.93	366.20 ± 8.02
thr	21.78 ± 0.88	18.03 ± 8.46
ala	98.54 ± 5.92	132.58 ± 7.21 **
pro	94.58 ± 5.26	47.18 ± 0.57 **
cys	1.04 ± 0.11	0.72 ± 0.11 *
tyr	5.05 ± 0.67	3.15 ± 5.60 **
val	24.60 ± 2.55	25.50 ± 5.15
met	3.09 ± 0.95	3.24 ± 0.15
lys	14.63 ± 1.25	7.23 ± 1.50 **
ile	18.10 ± 1.16	19.92 ± 4.15
leu	34.87 ± 1.18	32.34 ± 4.81
phe	51.34 ± 1.61	3.98 ± 0.96 **
Sum	982.11 ± 3.20	870.40 ± 2.23 **
EAA	168.42 ± 1.03	110.23 ± 4.43 **
HAA	332. 56 ± 8.61	268.6 ± 3.56 **
AAA	56.40 ± 8.61	7.13 ± 3.83 **
PCAA	434.52 ± 6.95	384.33 ± 5.73
NCAA	140.78 ± 1.81	138.60 ± 6.29

EAA, essential amino acids (ile, leu, lys, met, phe, thr, and val); HAA, hydrophobic amino acids (ala, val, iso, leu, tyr, phe, pro, meth, and cys); AAA, aromatic amino acids (phe and tyr); PCAA, positively charged amino acids (arg, his, and lys); NCAA, negatively charged amino acids (asp and glu). * *p* < 0.05 and ** *p* < 0.01 compared with the active fraction from meat.

**Table 2:** The DPPH radical scavenging, ACE-inhibitory, and α-amylase inhibitory activities of fractions purified from M-F and VM-F using HILIC (*n* = 3, mean ± standard deviation).

Origin	Fractions	DPPH Radical Scavenging Activity (%)	ACE-Inhibitory Activity (%)	α-Amylase Inhibitory Activity (%)
M-F	M-P1	14.73 ± 3.36	12.25± 1.67	10.25 ± 2.00
	M-P2	25.03 ± 3.11	15.26 ± 3.34	12.55 ± 0.62
	M-P3	20.75 ± 2.39	16.76 ± 3.12	15.26 ± 1.30
	M-P4	27.25 ±1.82	20.77 ± 0.85	15.76 ± 1.26
	M-P5	33.80 ± 2.93	23.07 ± 2.61	24.55 ± 0.29
	M-P6	91.87 ± 0.62	84.81 ± 0.35	56.15 ± 1.64
	M-P7	34.80 ± 1.22	28.20 ± 3.54	17.85 ± 2.77
	M-P8	32.60 ± 2.17	26.45 ± 1.90	16.45 ± 3.01
	M-P9	35.25 ± 1.40	25.85 ± 1.37	15.85 ± 2.27
VM-F	VM-P1	14.63 ± 3.38	12.50 ± 2.26	11.50 ± 2.46
	VM-P2	17.03 ± 2.07	14.56 ± 2.33	13.55 ± 2.61
	VM-P3	28.70 ± 0.99	20.56 ± 1.10	15.55 ± 1.36
	VM-P4	24.77 ± 3.71	23.85 ± 3.88	16.35 ± 1.34
	VM-P5	33.73 ± 1.47	25.80 ± 3.81	16.30 ± 1.74
	VM-P6	38.40 ± 1. 84	28.05 ± 2.80	18.05 ± 4.27
	VM-P7	82.60 ± 0.86	74.95 ± 1.24	53.85 ± 0.92
	VM-P8	35.60 ± 1.99	27.95 ± 0.76	26.95 ± 4.49
	VM-P9	29.93 ± 0.71	24.95 ± 0.85	24.96 ± 0.79
	VM-P10	28.03 ± 3.78	21.55 ± 1.64	11.56 ± 2.87
	VM-P11	32. 90 ± 0.86	27. 35 ± 3.36	17. 91 ± 1.70

**Table 3:** Docking energies for optimal conformation of two active peptides and ACE. YIAEDAER: Tyr-Ile-Ala-Glu-Asp-Ala-Glu-Arg; YSQLENEFDR: Tyr-Ser-Gln-Leu-Glu-Asn-Glu-Phe-Asp-Arg.

Peptides	- CDocker Energy (kcal/mol)	- CDocker Interaction Energy (kcal/mol)
YIAEDAER	174.672	130.72
YSQLENEFDR	193.884	175.07

## Supplementary Materials

The following are available online at http://www.mdpi.com/1660-3397/16/12/473/s1. Figure S1: Proximate compositions of the meat and visceral mass of *Nac*. (A) Fresh weight; (B) dry weight. Different letters indicate a significant difference between samples (*p* < 0.01); Figure S2: Molecular weight distribution of the active fraction from meat (A) and visceral mass (B) of *Nac*; Figure S3: MS/MS spectra of active peptides; (A) M-P6 from the meat and (B) VM-P7 from visceral mass of *Nac*. Figure S4: Visualization of *Neptunea arthritica cumingii*. (A) overall appearance, (B) soft body, (C) meat, and (D) visceral mass; Table S1: Pearson correlation coefficients (R) for various activity assays.
