# Supplementary material for: Two Novel Multi-Functional Peptides from Meat and Visceral Mass of Marine Snail Neptunea arthritica cumingii and Their Activities In Vitro and In Vivo
Source: Mar Drugs. 2018 Nov 27;16(12):473. doi: 10.3390/md16120473 (PMC6315844; doi:10.3390/md16120473)
Supplement: Supplementary file 1 [file marinedrugs-16-00473-s001.pdf]

Figure S1

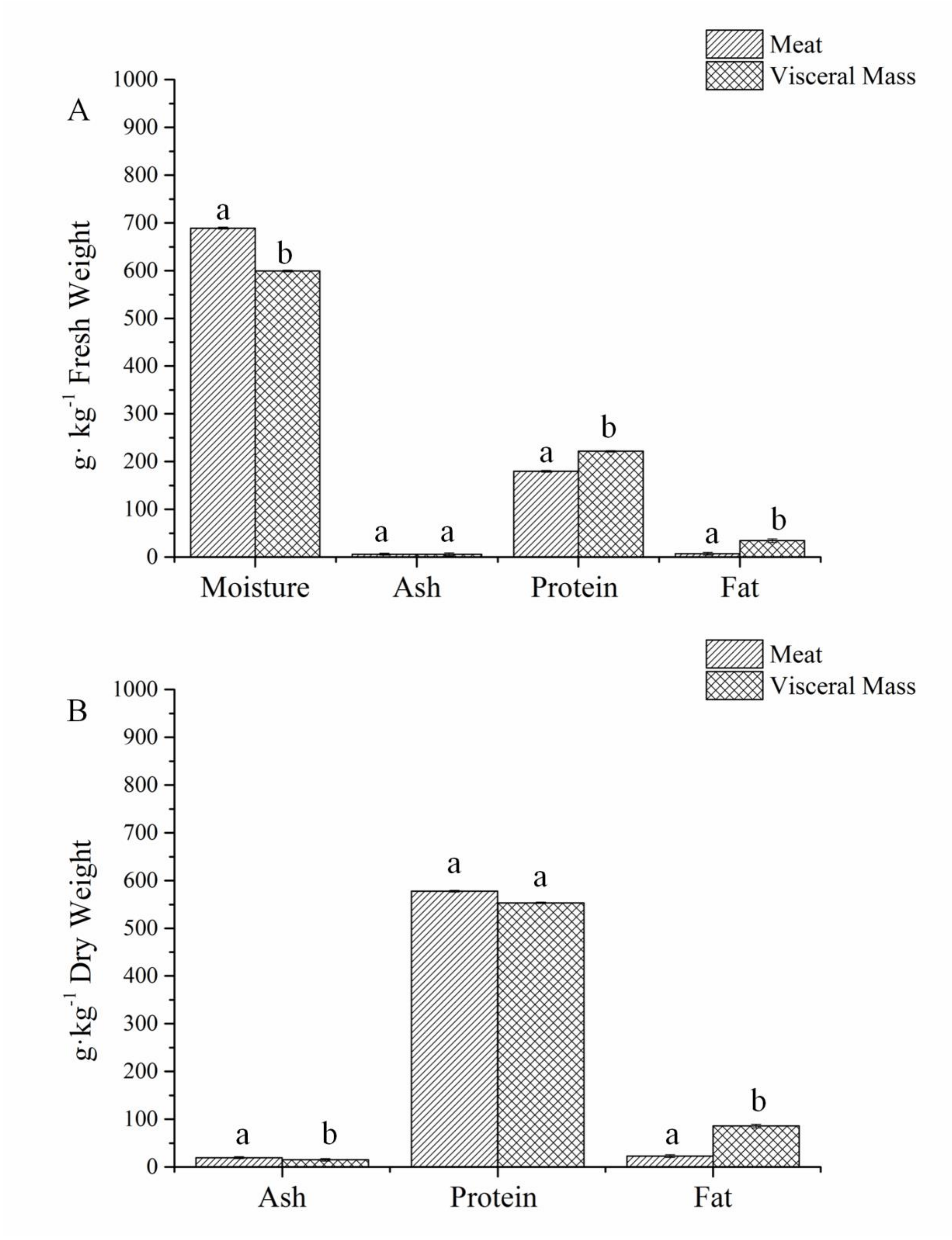
Meat
Visceral Mass
A
g· kg-1 Fresh Weight
1000
900
800
700
600
500
400
300
200
100
0
a
b
a
a
a
b
a
b
Moisture
Ash
Protein
Fat
Meat
Visceral Mass
B
g·kg-1 Dry Weight
1000
900
800
700
600
500
400
300
200
100
0
a
b
a
a
a
b
Ash
Protein
Fat

Figure S2

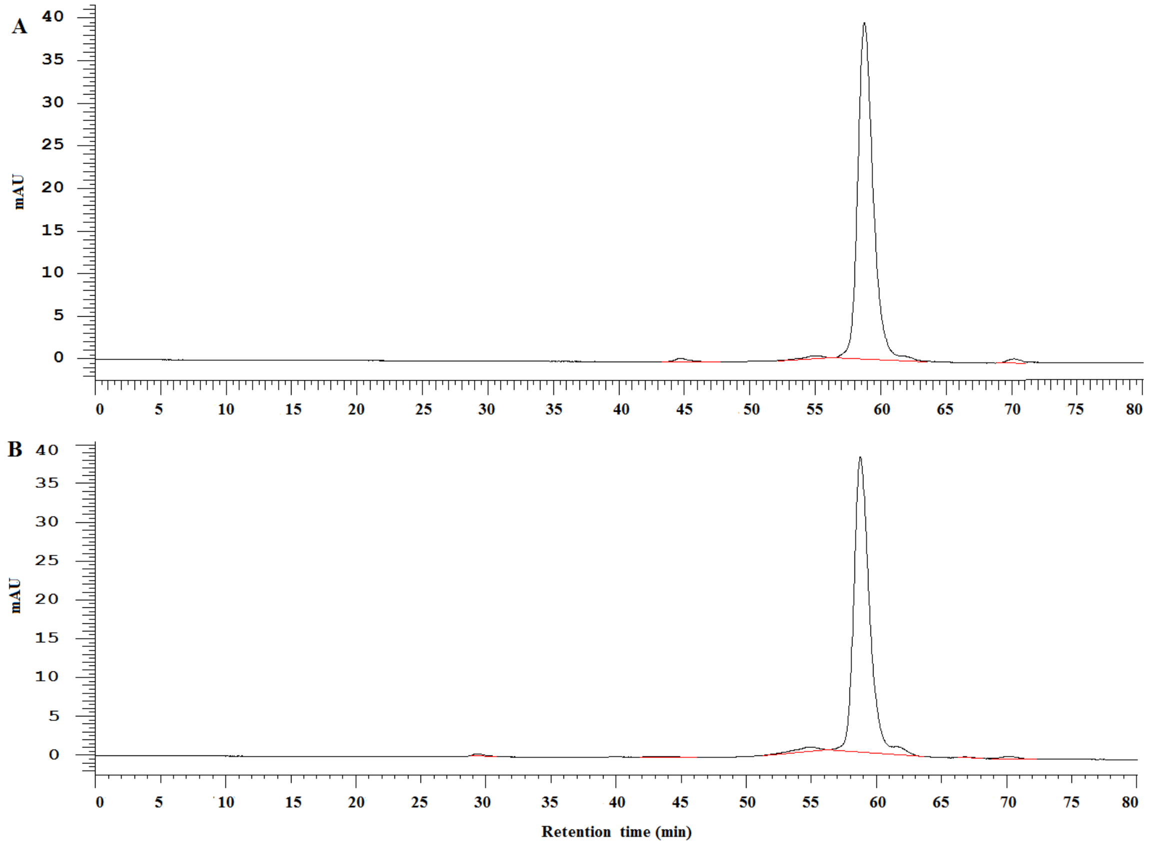

Figure S3

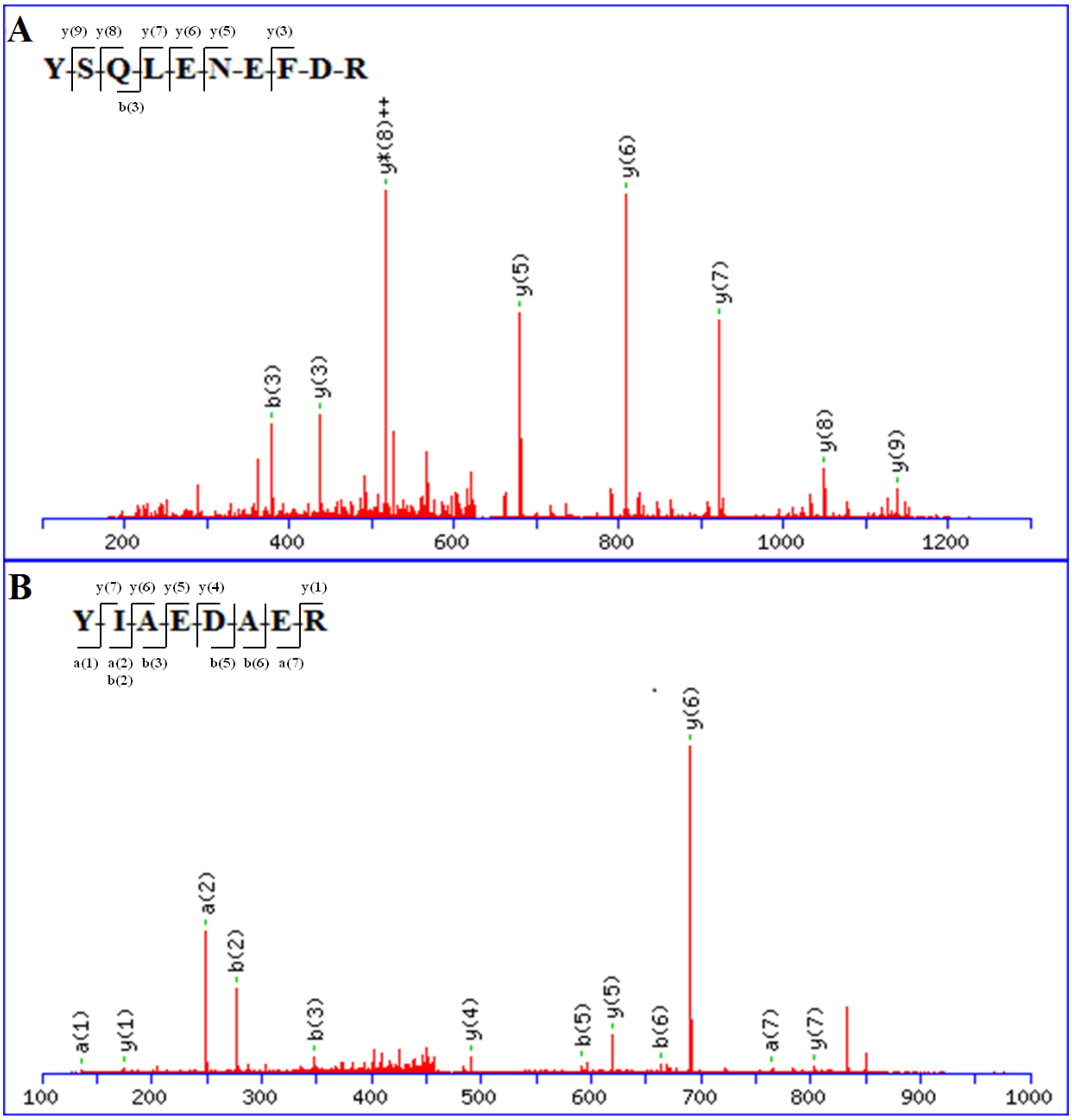
A
y(9) y(8) y(7) y(6) y(5) y(3)
Y-S-Q-L-E-N-E-F-D-R
b(3)
y*(8)++
y(6)
y(5)
y(7)
b(3)
y(3)
y(8)
y(9)
200
400
600
800
1000
1200
B
y(7) y(6) y(5) y(4) y(1)
Y-I-A-E-D-A-E-R
a(1) a(2) b(3) b(5) b(6) a(7)
b(2)
y(6)
a(2)
b(2)
a(1)
y(1)
b(3)
y(4)
b(5)
y(5)
b(6)
a(7)
y(7)
100
200
300
400
500
600
700
800
900
1000

Table S1 Pearson correlation coefficients (*R*) for various activity assays

Origin	Group	Absorbance	DPPH	ACE-inhibition	α-Amylase inhibition
Meat	Absorbance	--	0.625	0.759	0.796
	DPPH	0.625	--	0.859	0.766
	ACE inhibition	0.759	0.859	--	0.836
	α-Amylase inhibition	0.796	0.766	0.836	--
Visceral Mass	Absorbance	--	0.815	0.627	0.877
	DPPH	0.815	--	0.851	0.942
	ACE inhibition	0.627	0.851	--	0.812
	α-Amylase inhibition	0.877	0.942	0.812	--
